# Operationalising Article 13 of the Convention on the Rights of Persons with Disabilities the role of assistive technology in ensuring access to justice

**DOI:** 10.3389/fresc.2025.1650487

**Published:** 2025-10-30

**Authors:** Joanne McVeigh

**Affiliations:** ^1^ Department of Psychology, Maynooth University, Maynooth, Ireland; ^2^ Assisting Living & Learning (ALL) Institute, Maynooth University, Maynooth, Ireland; ^3^ The Bar of Ireland, The Law Library, Dublin, Ireland

**Keywords:** disability, access to justice, assistive technology, assistive products, Convention on the Rights of Persons with Disabilities

## Abstract

Access to justice is a determinant of the realisation of all other rights, including the right to health, employment, and education. As persons with disabilities experience increased discrimination and social exclusion and are at higher risk of violence than people without disabilities, it is crucial to ensure access to justice in both the civil and criminal legal spheres for people with disabilities. However, persons with disabilities experience multiple barriers at the macro/structural and individual levels to accessing justice. In light of the significance of access to justice for people with disabilities, and the multiple barriers to accessing justice experienced by those with disabilities, this perspective examines the importance of assistive technology in fulfilling the right to access justice. To fulfill the right of access to the justice system, assistive technologies must be more effectively harnessed to provide equitable access to justice for persons with disabilities.

## Introduction

Barriers to accessing justice are both a driver and outcome of poverty and disadvantage, whereby unmet justice needs may result in physical and mental health problems and decreased access to education, employment, and economic opportunities (1). However, persons with disabilities experience multiple barriers at the macro/structural and individual levels to accessing justice. These barriers include lack of information in accessible formats; lack of physical access to justice institutions and lack of accessible transport to such facilities; barriers to accessing legal support and representation; limitations on the exercise of legal capacity; paternalistic and negative attitudes in relation to the abilities of persons with disabilities; and lack of awareness and training amongst stakeholders in the legal profession (2).

Barriers to access to justice exacerbate the effects of socio-economic marginalisation experienced by persons with disabilities, such as poorer health and healthcare, lower quality of education, limited job opportunities, and barriers more broadly to participating in the community (3). For instance, if a person with a disability is denied the right to work and seeks a remedy from the justice system, but the justice system fails to provide physical, communication or other disability-related accommodations and/or explicitly discriminates against the individual, then the violation of the right to access justice also results in the infringement of the right to work (4).

It is also important to acknowledge intersectionality, whereby experiences of other identities such as gender, ethnicity and class influence individual experiences in the justice system (5). As emphasised by the OHCHR (6), specific challenges and mechanisms of exclusion in the justice system must be addressed for people with different forms of impairment such as Deaf persons and people with psychosocial or intellectual disabilities, in addition to persons with disabilities who are refugees, indigenous people, those living in rural areas, people living in poverty, intersex persons, and others who experience intersecting discrimination. For instance, with regards to the intersection between gender and disability, direct discrimination arises when women with psychosocial or intellectual disabilities who are victims of violence provide testimonies that are disregarded in court proceedings due to legal capacity, therefore impeding effective legal remedies and access to justice for such women (7).

Furthermore, disability interacts with other social characteristics that may lead to an increased risk of discrimination and/or violent crime, for example, amongst women and girls with disabilities (8, 9), children with disabilities (10, 11), and people with disabilities of diverse sexual orientations and gender identities (12, 13). Such populations may therefore be more likely to require access to justice while also experiencing specific mechanisms of exclusion in the justice system.

As persons with disabilities experience discrimination and exclusion across all areas of life, including employment, education, and healthcare (14) and are at higher risk of violence than people without disabilities (15), it crucial to ensure access to justice in both the civil and criminal legal spheres for people with disabilities. As noted by Beqiraj (3), as a human right in itself and as a prerequisite to the vindication of all other rights, access to justice is particularly important for persons with disabilities as a mechanism to overcome discrimination and violence.

Access to justice is therefore a *sine qua non* for the realisation of all other rights. So too, conversely, the fulfillment of other rights for persons with disabilities enables access to justice. As asserted by Ortoleva (4), the fulfillment of other human rights can positively or adversely influence the ability of persons with disabilities to access justice, including accessible transportation so that people with disabilities can travel to courts and police stations, access to education to enable understanding of the justice system, and political participation so that persons with disabilities can run for office or vote for political candidates who enable access to justice for people with disabilities.

Importantly, this also includes the right to assistive technologies (AT), without which the right to justice in civil and criminal law cannot be upheld. Accordingly, a report by G3ict and the International Disability Alliance has called for the use of technology to increase access to justice for people with disabilities (16). The intersectionality of disability and assistive technology needs must also be considered for those who may experience intersectional types of exclusion in addition to increased barriers to accessing AT (17). Importantly, particular populations may therefore be more likely to require access to justice while also experiencing specific mechanisms of exclusion in the justice system and increased barriers to accessing AT.

In view of the significance of access to justice for people with disabilities, and the multiple barriers to accessing justice experienced by those with disabilities, this perspective examines access to assistive technologies as a mechanism to operationalise Article 13 of the United Nations Convention on the Rights of Persons with Disabilities (UNCRPD). First, a case study is presented to illustrate the importance of AT in enabling access to justice. While the following case study presents a fictional account, it is based on a number of real-life experiences of access to AT in the legal sector [for example, please see National Association of the Deaf (18) and Magistrates' Association (19)].

## Case study

Isabella has worked as a manager for a marketing organisation for fifteen years. As a result of a recent incident, she has incurred significant hearing loss. Having consulted with a medical specialist, Isabella believes that a number of measures could be taken by her organisation to allow her to continue in her role, without posing a disproportionate burden on her employer. However, when Isabella tries to return to work and to discuss her additional needs with management, they dismiss her from her role and refuse to provide reasonable accommodation.

To compel her employer to consider reasonable accommodation, Isabella has sought legal advice, and the case is being heard by the State's employment tribunal. Isabella has asked to avail of a hearing loop system for the duration of the legal proceedings. However, she has been informed that this system will not be available. Isabella has expressed concern that she will therefore be unable to properly engage with the legal proceedings. However, her legal team tell her that they will assist her by updating her at intervals throughout the tribunal hearing.

However, Isabella is unable to adequately hear the proceedings, including the testimony of her employer, questions being asked by the tribunal, and questions being posed to her by her own legal team and by counsel for the defendant. The tribunal ultimately finds in favour of the defendant organisation. Isabella believes that not only has she been subjected to unfair treatment by her organisation, she has been subject to secondary victimisation by the legal system and has therefore been hindered in redressing the discriminatory dismissal by her employer and in accessing justice.

## Article 13 UNCRPD: access to justice

Article 13(1) UNCRPD on “Access to Justice” calls for States Parties to:

“…ensure effective access to justice for persons with disabilities on an equal basis with others, including through the provision of procedural and age-appropriate accommodations, in order to facilitate their effective role as direct and indirect participants, including as witnesses, in all legal proceedings, including at investigative and other preliminary stages”.

Article 13 therefore sets out extensive, wide-ranging obligations on States Parties to provide access to justice for persons with disabilities as participants in legal proceedings and as professionals in the justice system (20).

As emphasised in UN General Comment No. 1, access to justice is contingent on the recognition of the right of persons with disabilities to legal capacity and to be recognised as persons with equal status in courts and tribunals within the justice system (21). Importantly, this requires not only the development and enactment of inclusive and rights-based legislation but also *judicial interpretation* of legislation that conveys full personhood on persons with disabilities and that is non-paternalistic in its approach (22, 23).

The importance of appropriate training within the justice system is affirmed in Article 13(2) UNCRPD:

“In order to help to ensure effective access to justice for persons with disabilities, States Parties shall promote appropriate training for those working in the field of administration of justice, including police and prison staff”.

As stipulated in General Comment No. 1, the judiciary must therefore receive training to ensure awareness of their obligation to uphold the legal capacity of persons with disabilities; such training and awareness-raising must also be provided to other stakeholders within the justice system such as police officers, social workers, and other first responders (21). This is reiterated in Principle 10 of the International Principles and Guidelines on Access to Justice for Persons with Disabilities, which provides: *“*All those working in the justice system must be provided with awareness-raising and training programmes addressing the rights of persons with disabilities, in particular in the context of access to justice” (2).

As emphasised by O'Mahony (24), education and awareness programmes for people with disabilities, the wider population, central stakeholders in the justice system, and across the legal community are needed to ensure knowledge and understanding of access to justice and Article 13. Notably, however, those working in and engaging with the justice system can also provide valuable perspectives on ways to improve access to justice. It is therefore important to avail of the knowledge and expertise of court staff and external stakeholders, such as litigants, judges, clerks, legal counsel and law firms, to gain insight into how courts can be made more accessible (25).

## Importance of assistive technology in ensuring access to justice

The right to access justice is therefore stipulated in the UNCRPD as a discrete provision in Article 13. However, the interrelatedness of all human rights, as stated in the Preamble of the UNCRPD, implies that other provisions in the convention must be acknowledged when interpreting Article 13 and that other UNCRPD provisions, beyond Article 13, are relevant to access to justice (5).

In this regard, Articles 13 must be read in conjunction with the General Obligation set out in Article 4(1)(g), requiring States Parties to “…promote the availability and use of new technologies, including information and communications technologies, mobility aids, devices and assistive technologies…”. Furthermore, Article 4(1)(f) affirms the importance of universal design, calling for goods, services, equipment and facilities that “require the minimum possible adaptation and the least cost to meet the specific needs of a person with disabilities, to promote their availability and use, and to promote universal design in the development of standards and guidelines”.

Similarly, as highlighted by Flynn (26), while non-discrimination is not explicitly stated in Article 13, it is implied in the terminology “on an equal basis”, and, when read in conjunction with other provisions, is set out in Article 3 (General Principles), Article 4 (General Obligations), and Article 5 (Equality and Non-Discrimination), the latter article specifying the obligation to provide reasonable accommodation to promote equality and prevent discrimination.

The obligation of reasonable accommodation, although not explicitly mentioned in Article 13, is therefore applicable with respect to access to justice (20). As reasonable accommodation incorporates assistive technologies, this implies that the right to access justice is interdependent with the right to access AT. For example, assistive technologies such as screen readers and automatic transcriptions can enable participation in court for persons with disabilities (27).

Article 13 may also be interpreted with regards to Article 32 on “International Cooperation”, which at (1)(d) calls on States Parties to provide “as appropriate, technical and economic assistance, including by facilitating access to and sharing of accessible and assistive technologies, and through the transfer of technologies”. As affirmed in the Global Report on Assistive Technology, in relation to Article 32 [(28), p. xiii]:

“Such cooperation can support efforts in areas of research, policies, regulations, fair pricing, market shaping, product development, technology transfer, manufacturing, procurement, supply, service provision and human resources. International cooperation is essential to reducing inequity and progressively achieving universal access to assistive technology—and leaving no one behind”.

Importantly, international cooperation can therefore support market shaping, whereby market shaping aims to improve a market's outcomes, such as equitable access to high-quality and low-cost assistive technology, by addressing the underpinning causes of failures in the market and disparities in demand and supply (29). Indeed, equitable access to AT is dependent on the sector employing a considerably stronger systems thinking and market shaping approach (30). As emphasised by MacLachlan et al. [(31), p. 5], although “[t]he history of AT provision in many countries, especially low- and middle-income countries, is one of small-scale local providers manufacturing products of varying degrees of quality and often at a restricted price range”, it is crucial “to envisage an effective way of scaling these production and provision enterprises to the level required to close the gap between available and required AT, which is both affordable and of acceptable quality”. Accordingly, one of the main levers by which international cooperation may support equitable access to AT—and therefore access to justice—is through market shaping.

Article 13 may also be interpreted with respect to Article 20(b) UNCRPD on “Personal Mobility”, which provides for “…access by persons with disabilities to quality mobility aids, devices, assistive technologies and forms of live assistance and intermediaries, including by making them available at affordable cost”. In this respect, it is important to read the right to access justice, as provided for in Article 13, in conjunction *inter alia* with Articles 4, 5, 20, and 32, which set out the obligations on States Parties to fulfill the right to assistive technologies and to espouse principles of universal design.

Importantly, universal design or the adaptation of products and environments and the provision of assistive technologies can mitigate social exclusion and support accessibility for persons with disabilities (28) and are therefore imperative to enable access to the justice system. Indeed, assistive technology in the courtroom can assist not only persons with disabilities but also people without disabilities to meaningfully engage with proceedings, especially when courts employ universal design principles, such as real-time captioning of proceedings on a display monitor for judges, jurors, and legal counsel, who for example may not have heard or misheard a witness's last statement (32). As asserted by MacLachlan (33):

“By adopting principles of both universal design and being sensitive to individuals’ needs for reasonable accommodations, AAC and AT; many more people can be included, *the centre can hold, and hold more firmly*, the gyre need not widen but can bring in others from the margins too” [emphasis added].

The provision of AT is therefore key to the realisation of the rights enshrined in the UNCRPD including access to justice (34). Relatedly, the SDGs call for access to justice for all and inclusive institutions at all levels, as a stand-alone goal in SDG 16. However, none of the SDGs can be achieved equitably without ensuring the availability of assistive products (35). AT is therefore key to equitably realising SDG 16, ensuring that no-one is left behind and enabling access to justice for all.

## Conclusion

Access of persons with disabilities to the civil justice system is vital to address and redress discrimination and social exclusion experienced by people with disabilities across all domains of life. As persons with disabilities are also at higher risk of violence than people without disabilities (15), access to justice in the criminal legal sphere is also imperative. Otherwise, persons with disabilities risk being subject to secondary victimisation, namely victimisation that arises not as a direct outcome of the criminal offence but due to the response of public or private institutions and the person's interaction with the criminal justice system (36, 37).

The intersectionality of disability and assistive technology needs must also be considered for those who may experience intersectional types of exclusion in addition to increased barriers to accessing AT (17), whereby intersectionality may impact access to AT and more broadly the need for, and experiences of, access to the justice system.

Fulfilling Article 13 UNCRPD is a prerequisite to the realisation of all other rights; yet persons with disabilities continue to experience multiple barriers at the macro/structural and individual levels to accessing the justice system. In this regard, it is crucial for States to collect and analyse disaggregated data in relation to violations of the rights of persons with disabilities and the extent to which the justice system is providing access to justice, including access to a fair trial and effective remedies (6). This also requires the systematic collection of data on AT-needs, use of AT, and facilitators and barriers to accessing AT in the justice system.

A recent Opinion by the Consultative Council of European Judges [(38), p. 3] emphasises that “societal use of technology will continue to develop. Courts and judiciaries should keep pace with such developments”. The Opinion further emphasises “the importance of developing and using technology in ways that maintain and, where possible, enhance the fundamental principles of the rule of law”. Upholding the rule of law, by ensuring access to justice for all, will require operationalising Article 13 UNCRPD through the harnessing of assistive technologies.

## Data Availability

The original contributions presented in the study are included in the article/Supplementary Material. Further inquiries can be directed to the corresponding author.
